# Quantitative analysis of cells encapsulated in a scaffold

**DOI:** 10.1016/j.mex.2020.101146

**Published:** 2020-11-24

**Authors:** Marfa N. Egorikhina, Diana Ya Aleynik, Yulia P. Rubtsova, Irina N. Charykova

**Affiliations:** Federal State Budgetary Educational Institution of Higher Education Privolzhsky Research Medical University of the Ministry of Health of the Russian Federation, Nizhny Novgorod, Russian Federation

**Keywords:** Scaffold, Hydrogel, Cell nuclei, Cell viability, Proliferation, Fluorescence microscopy

## Abstract

One of the urgent problems arising while carrying out research in the field of scaffold technology is achieving an objective, direct, quantitative analysis of cells cultivated within a scaffold; one which allows characterization of the density distribution of the cells, their viability and their proliferative activity when encapsulated within the scaffold. This problem is associated with the peculiarities of cell cultivation in the three-dimensional structure of scaffolds, including limitations imposed on the possibility of direct cell counting using light microscopy. Also, most scaffolds are opaque, so this generally excludes methods of quantitative analysis using light microscopy. There are methods for the quantitative analysis of cells in a scaffold based on the assessment of their metabolic activity (for example: MTT test). However, these methods are indirect and can result in significant errors. This is due to differences in the metabolic activity of the cells, for example, in different phases of mitosis. Methods based on direct counting of the number of cells isolated from the scaffold are also characterized by a high degree of error that is associated with the loss of cells during the destruction of the scaffold. We describe in detail a method that allows the direct quantitation of cells within a scaffold. Modifications of the method make it possible both to analyze the proliferative activity of cells cultivated in a scaffold and to assess their viability and density distribution in the three-dimensional structure.

• Direct rather than indirect analysis of the number of cells in the scaffold by counting the number of nuclei.
• Carrying out research without destroying the scaffold structure.
• Carrying out research without additional preliminary preparation of samples before staining.

## Graphical abstract

**Figure:**
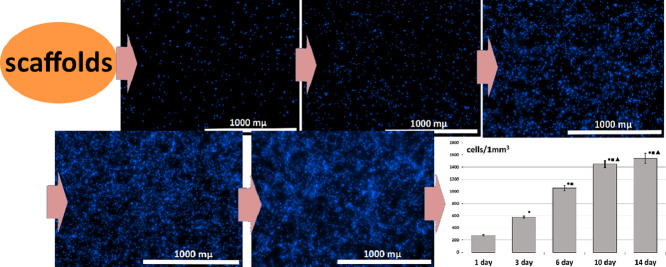

Specifications table

**Table:** 

Subject Area:	• *Medicine*
More specific subject area:	*• Tissue engineering* *• Scaffold technologies*
Method name:	*Quantitative analysis of cells in a scaffold using a wide-field fluorescence microscopy*
Name and reference of original method:	*[1] M.N. Egorikhina, I.N. Charykova, D.Y. Alejnik, Patent №2675376 The Russian Federation, Int. Cl. G01N 33/52 Method of quantitative analysis of cellular components of scaffold. Application: 2017125696, 17.07.2017, Bull. 35. (2018).* *[2] M.N. Egorikhina, D.Y. Aleynik, Y.P. Rubtsova, G.Y. Levin, I.N. Charykova, L.L. Semenycheva, M.L. Bugrova, E.A. Zakharychev, Hydrogel scaffolds based on blood plasma cryoprecipitate and collagen derived from various sources: Structural, mechanical and biological characteristics, Bioact. Mater. 4 (2019) 334–345. doi:10.1016/j.bioactmat.2019.10.003.*
Resource availability:	https://bioline.ru/catalog/369/1073/ https://www.biotek.com/products/imaging-microscopy-cell-imaging-multi-mode-readers/cytation-5-cell-imaging-multi-mode-reader/

## Method details

 

## Quantitative analysis of cells encapsulated in a scaffold

Quantitative analysis of cells encapsulated in a scaffold can be carried out as follows:

I. Staining of the sample

1. A scaffold fragment of at least 0.64 cm^2^ is placed in a 24-well fluorescence microscopy plate with opaque side walls (for example, a Black VisiplateTM TC plate (Wallac Oy, Finland)).
2. Then, *in vivo* staining of the cell nuclei within the scaffold is carried out using Hoechst 33342 fluorochrome, which is highly specific for double-stranded DNA molecules. To each well of the plate containing a scaffold fragment and 2 ml of culture medium (the type depending on the cells being cultured in the scaffold, for example, DMEM or α-MEM containing 10% fetal calf serum), is added 1 µl of Hoechst 33342 (USA) solution at a concentration of 10 µg / ml. The plate is then incubated for 30 min at 37^0^ С.
3. After incubation, the dye medium in which the scaffold fragment was incubated is removed. The stained fragment of the scaffold is washed twice with phosphate buffer (PBS). To do this, we add 2 ml of PBS to the well with the scaffold fragment. We incubate for 10 min at room temperature. We remove the PBS from the well. We repeat the washing cycle again.
4. Then we add from 0.3 ml up to 1 ml of phosphate buffer to the scaffold fragment to prevent the sample from drying out during subsequent analysis.

II. Data visualization and recording

**Fig. 1:**
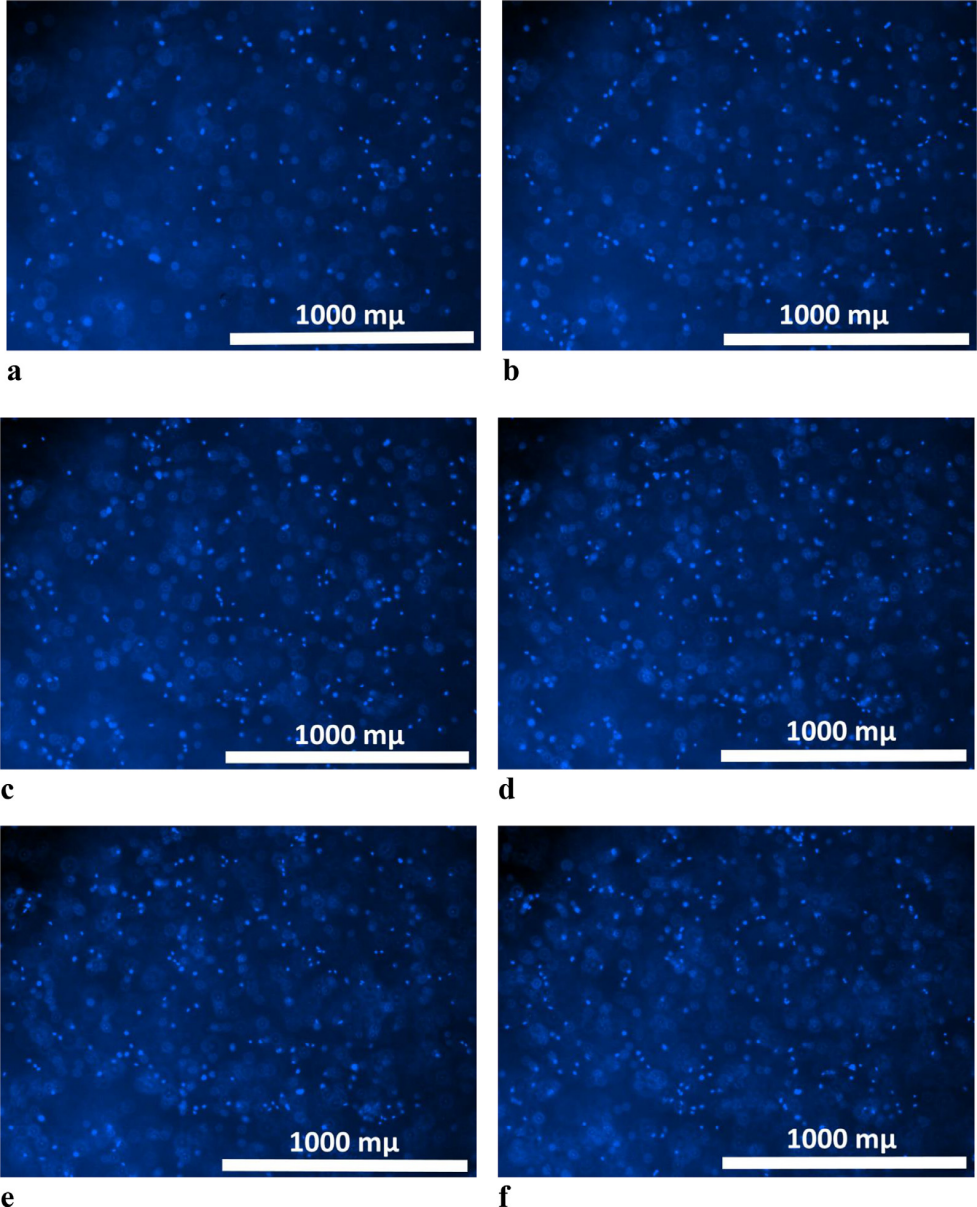
A series of sequentially taken images with a 4x lens moving along the Z axis (a – 0 µm; b – 106 µm; c – 212 µm; d – 318 µm; e – 424 µm; f –530 µm) of a hydrogel scaffold with encapsulated mesenchymal stem cells. Cell nuclei were stained with Hoechst 33342.

1. The plate with the scaffold fragments is transferred to a facility that can conduct fluorescence microscopy and which is equipped with the Z-stack shooting function. We implement the described method using a Cytation ™ 5 imager with Gen5 Image software (BioTek, USA). The Cytation ™ 5 imager functions like a wide-field fluorescence microscope but with the ability to implement the Z-stack function. While using the Z-stack function, the lens is moved along the Z-axis and takes multiple images (Fig. 1). It detects only those areas that are in focus, and synthesizes them into a fully focused image (stitched image) (Fig. 2a).

**Fig. 2:**
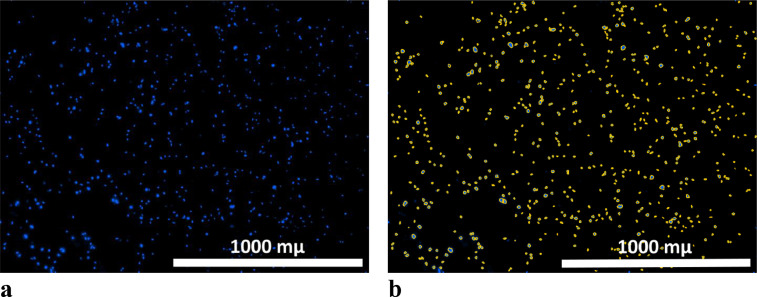
(a) Stitched Z-stack image of one field of view (the depth along the Z axis is 530 µm; 4x objective lens) of a hydrogel scaffold with encapsulated mesenchymal stem cells. Cell nuclei were stained with Hoechst 33342. (b) The same image with a “mask” applied when using a fluorescence glow intensity threshold filter (the analysis took into account only objects with a glow intensity of more than 7000) and an object area filter (during the analysis, only objects with an area less than 30 µm were taken into account).
2. Imaging is carried out using a fluorescence channel corresponding to the dye (excitation wavelength of 377 nm and emission wavelength of 477 nm) in 5 or more fields of view using a fourfold or tenfold lens. For each selected field of view, layer-by-layer shooting is carried out along the Z-axis to a given depth of no more than 530 µm, followed by image stitching (Z-stack). The result is a series of stitched Z-stack images: one stitched Z-stack image for each field of view.

III. Image processing

1. The stitched Z-stack images, which were obtained according to the methosd specified in item II, were processed using Gen 5 Image software. The number of cell nuclei was counted on each stitched Z-stack image. While processing, a fluorescence glow intensity threshold filter was used (during the analysis, only those objects with a luminescent glow intensity of more than 7000 were taken into account), together with an object area filter (during the analysis, only objects with areas of less than 30 µm were taken into account).

The use of these filters allows us to reduce errors in the quantitative analysis. Thus, objects with a luminescence intensity below the selected brightness threshold are referred to as lighter objects and are excluded from the analysis (for example: stained cell nuclei that lie in deeper layers of the scaffold than the layer under examination).

The application of the fluorescence glow intensity threshold filter also makes it possible to separate numbers of closely spaced cell nuclei. At their centers, nuclei exhibit more intense luminescence than in their peripheries, thus, the objects can be separated according to the intensity of the luminescence since, while carrying out the analysis, only those objects with a brightness higher than the threshold are taken into account. These represent the central parts of the nuclei.

The application of the object area filter allows us to avoid our analysis taking into account objects with areas greater than a predetermined value, which makes it possible to exclude objects that are not related to the cell nuclei (for example: dye residues) or clusters of closely spaced cells that would not have been separated when applying the previous filter.

When choosing threshold values while working with previously unexamined samples (for which the filter parameters may vary), it is necessary to confirm that all visualized objects that are cell nuclei are included in the analysis. This can be done by applying the “mask” function and visually assessing whether all visible cell nuclei have been taken into account in the quantitative analysis (Fig. 2b). If there are unaccounted cell nuclei, or objects that are not related to cell nuclei, but which have been included in the analysis, then this indicates that it is necessary to select new threshold values of the filters to be used for further analysis.

IV. Quantitative analysis

1. The total number of cell nuclei in each of 10 or more of stitched Z-stack images is estimated.
2. The average number of cells in 10 images or more of stitched Z-stack images is also estimated..
3. The number of cells in 1mm^3^ of the scaffold is calculated according to the formula:
  $$K=\frac{N}{B*C*D}*10^{-9},$$

where K is the number of cells in 1mm^3^;

N is the average number of cells in 10 images or more of stitched Z-stack images (IV.2);

B is the size of the analyzed field of view in µm along the X-axis when using a lens with a 4-fold or 10-fold magnification;

С is the size of the analyzed field of view in µm along the Y axis when using a lens with a 4-fold or 10-fold magnification;

D is the size of the analyzed field of view in µm along the Z axis when using a lens with a 4-fold or 10-fold magnification;

10^−9^ is a coefficient that determines the conversion of µm^3^ to mm^3^.

As a result of the analysis performed, we can determine the number of cells in 1 mm^3^ of the scaffold, which allows us to characterize the density of distribution of the cells in the scaffold.

## Analysis of the proliferative activity of cells cultivated in a scaffold

Using the method described above for the quantitative analysis of the cells encapsulated in a scaffold, it is possible to analyze the proliferative activity of cells being cultured in a scaffold. To characterize such proliferative activity, we suggest the following algorithm:

1. After its production the scaffold containing cells is cultured under standard conditions appropriate to the type of cells encapsulated. For example, if mesenchymal stem cells are encapsulated in the scaffold, then it would be cultured in an appropriate growth medium in a CO_2_ incubator (+37°C, in a humidified atmosphere with 5% СО_2_). The duration of cultivation is determined depending on the objectives of the research. For example, up to 14 days following the formation of the scaffold.
2. At sample points, determined depending on the objectives of the research (for example, 1, 3, 7, 10, 14 days), a fragment of at least 0.64 cm^2^ is separated from the cultivated scaffold. A quantitative analysis of the cells in the isolated fragment is then carried out according the method described above.
3. A comparative analysis of the number of cell nuclei in the scaffold can then be carried for each sample in the time frame.

The method allows assessment of the dynamics of changes in the number of cells encapsulated in the scaffold during its cultivation and characterization of the proliferative activity of the cells.

## Evaluation of the viability of cells encapsulated in a scaffold

Using the algorithm described above for the quantitative analysis of cells encapsulated in a scaffold, it is possible to assess the viability of these cells. For this, it is necessary to use two fluorochrome nuclear dyes, one of which stains the nuclei of both living and dead cells, while the second dye stains the nuclei of the dead cells only.

The method is carried out in accordance with that described above "Quantitative analysis of cells encapsulated in a scaffold" with the following modifications:

1. Par. I.2 is changed: Then, intravital staining of the cell nuclei in the scaffold is carried out using Hoechst 33342 fluorochrome (USA) and PROTM3 Ready FlowTM fluorochrome (invitrigen by Thermo Fisher Scientific, USA), allowing identification of dead cell nuclei. To do this, 1 µl of Hoechst 33342 (USA) solution at a concentration of 10 µl/ml is added to each well of the plate containing the scaffold fragments in 2 ml of culture medium (depending on the cells being cultured in the scaffold, for example, DMEM or α-MEM containing 10% fetal calf serum). The plate is incubated for 15 min at 37^0^С. Then 4 drops of TO-PROTM3 Ready FlowTM fluorochrome are added to each well containing a test sample. The plate is incubated for a further 15 min at 37^0^С.
2. Par. II.2 is modified: Imaging is carried out using fluorescence channels corresponding to the dyes. To visualize the nuclei of cells stained with the Hoechst 33342 fluorochrome (excitation wavelength of 377nm and emission wavelength of 477nm), the “DAPI” fluorescence channel is used. To visualize the nuclei of cells stained with TO-PROTM3 Ready FlowTM fluorochrome (excitation wavelength of 586 nm and emission wavelength of 647 nm), the “TaxasRed” fluorescence channel is used. In each field of view, sequential imaging is carried out using the DAPI channel followed by the TaxasRed channel. Imaging in each field of view (5 or more fields of view) is carried out using a lens with 4-fold or 10-fold magnification. In each selected field of view (on each channel), layer-by-layer imaging is performed along the Z-axis at a given depth of no more than 530 µm, followed by stitching of the images (Z-stack). The result is a series of stitched Z-stack images: each pair of stitched Z-stack images corresponding respectively to the DAPI and TaxasRed channels for a particular field of view.
3. After paragraphs III-IV have been completed, we have the data on the total number of cells in 1mm^3^ and the data on the number of dead cells in 1mm^3^ of the scaffold. If required, it is therefore possible to calculate the percentage of dead cells per 1mm^3^ of the scaffold.

## Method validation

1. The presented method is quite sensitive and allows accurate analysis of the number of cell nuclei in the scaffold [1]. To validate the method for counting numbers of cells, two hydrogel fibrin-collagen scaffolds containing human dermal fibroblasts were taken with a 2-fold difference in the number of cells (Var 1 – initial cell concentration 90 cells / mm^3^; Var 2 – 180 cells / mm^3^ – calculated values) and a relatively uniform distribution cells throughout the entire volume of the scaffold. The scaffolds were cultured for 3 days in DMEM growth medium containing 10% of fetal calf serum, in an incubator with a 5% CO_2_ concentration and a temperature of 37^0^С. Then a quantitative analysis of the cells encapsulated in the scaffold was carried out in accordance with the method described. It was shown that both data samples had a mean square deviation (Ϭ) less than 16% of the sample mean, and the standard error of the mean (m) did not exceed 5% from the sample mean (Table 1). The one-dimensional probability distribution is shown in the swing charts (Fig. 3). After three days of cultivation in the Var 1 scaffold, the number of cells had increased by 3 times; in the Var 2 scaffold, the number of cells increased by 3.5 times (Table 1). At the same time, the concentration of cells in the Var 2 scaffold was 2.3 times higher than in the Var 1 scaffold. Thus, the presence of the initially specified difference in the number of cells between the Var 1 and Var 2 scaffolds after cultivation indicates that the cells have proliferated at a comparable rate in both scaffolds.

**Table 1:** Quantitative analysis of cells encapsulated in a scaffold. Statistical characteristics of samples.

Field of view №	Var 1	Var 2
	number of cell nuclei (pcs / мм^3^)	number of cell nuclei (pcs / мм^3^)
1	301.73	645.38
2	250.05	648.17
3	203.95	648.87
4	238.87	637.00
5	308.02	609.06
6	276.59	553.88
7	321.99	602.07
8	213.03	681.70
9	273.10	662.84
10	299.64	743.86
Average (M)	**268.70**	**643.28**
mean square deviation (Ϭ)	40.89	50.48
standard error of the mean (m)	**12.94**	**15.98**

**Fig. 3:**
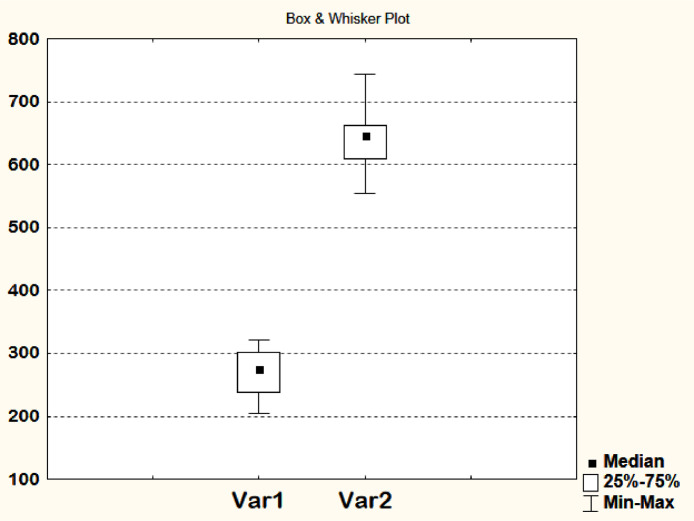
Swing chart of the quantitative analysis data for the Var 1 and Var 2 scaffolds after cultivation (statistical analysis was carried out using STATISTICA 6.0 software).
2. The presented technique was used in an experimental study to assess the proliferative activity of cells cultivated in scaffolds. 10 scaffolds of a type used to replace skin defects were studied, i.e. scaffolds based on natural biopolymers with encapsulated mesenchymal stem cells. The scaffolds were cultivated for 14 days in a CO_2_ incubator with 5% CO_2_ at a temperature of 37°C with a change of growth medium every 2–3 days. The number of cells was determined on days 1, 3, 6, 10 and 14 after the formation of the scaffold (day 0) according to the method described above (Analysis of the proliferative activity of cells cultivated in a scaffold). The research results were processed by methods involving nonparametric statistics, using the Wilcoxon pairwise comparison test and applying the STATISTICA 6.0 software package. It was shown that during the 10 days of cultivation within the scaffold, the cells maintained high proliferative activity (Fig. 4). However, by the 14th day, proliferative activity was decreasing [2].

**Fig. 4:**
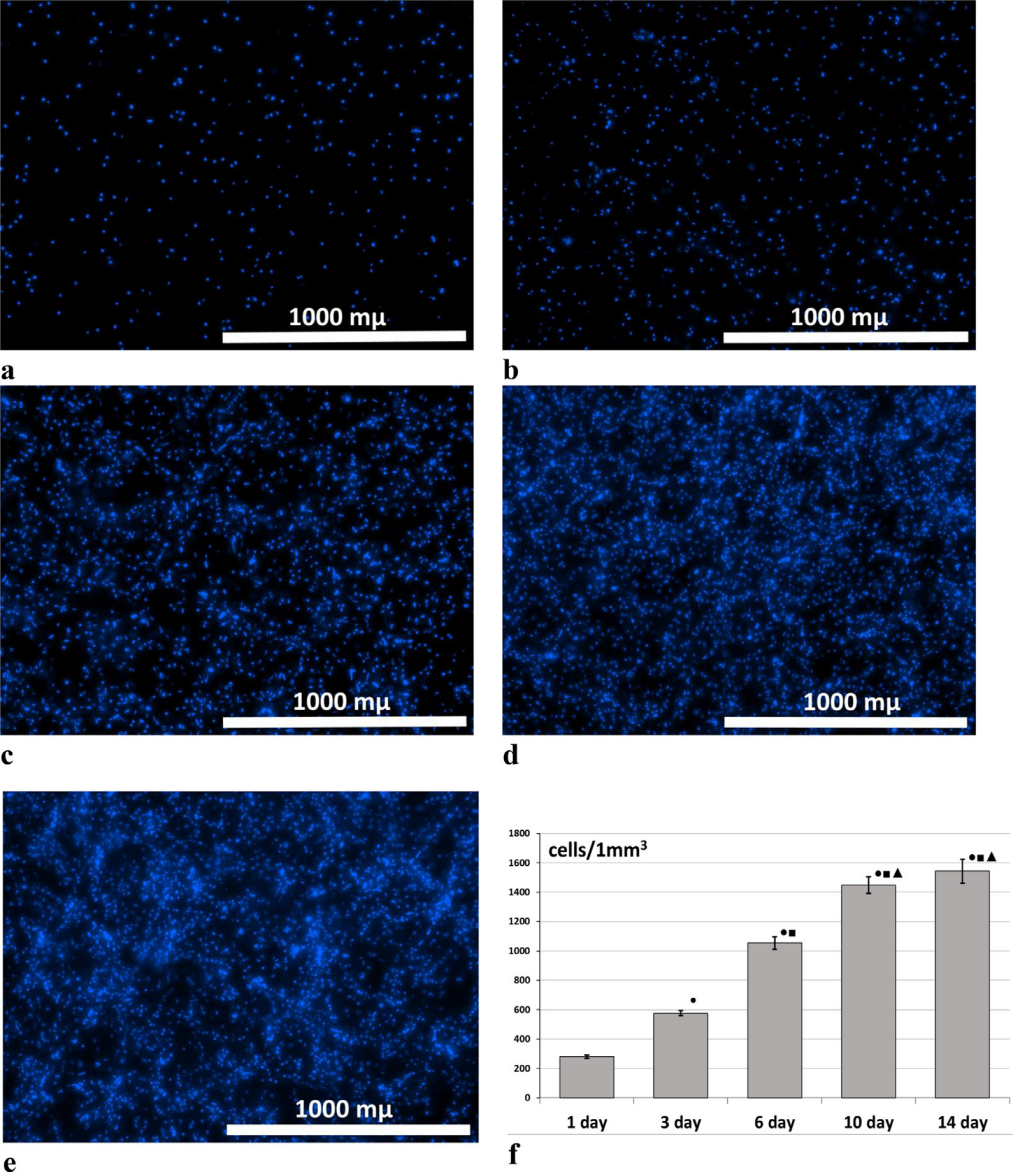
Analysis of proliferative activity of cells cultured in a scaffold. (a–e) Representative stitched Z-stack images. The cell nuclei are stained with Hoechst 33342, observed at 4x magnification. (a) day 1, (b) day 3, (c) day 6, (d) day 10, (e) day 14. (f) Results of analysis of the proliferative activity of cells cultured in the scaffold. Note: ● – p˂0.05 comparison with day 1, ■ – comparison with day 3, ▲ – comparison with day 6, ♦ – comparison with day 10, Wilcoxon test.
  The described method has also shown its viability in other studies related to the quantitative analysis of cells in scaffolds and the assessment of their proliferative activity [3].
3. For a comparative assessment of the density of distribution and viability of cells in two types of scaffold differing in their type of collagen (3 samples with collagen № 1 and 3 samples with collagen № 2), we used the above method (Assessment of the viability of cells encapsulated in a scaffold) using two fluorescent dyes, Hoechst 33342 and TO-PROTM3 Ready FlowTM. To do this, with a help of a template, fragments each with an area of 0.64 cm^2^ were isolated from the samples being studied. The number of cells was determined by counting the nuclei 3 hours after the formation of the scaffolds. After the first fragment had been isolated, the scaffolds were cultured under standard conditions. After 72 hours, further fragments with the area of 0.64 сm^2^ were removed and the number of cell nuclei was counted. To identify and count the number of cell nuclei, as before, we used Hoechst 3334 (USA), having an excitation wavelength of 377 nm and emission wavelength of 477 nm. To identify and count dead cells, the samples were stained with TO-PROTM3 Ready FlowTM fluorochrome (invitrigen by Thermo Fisher Scientific, USA), having an excitation wavelength of 586 nm and emission wavelength of 647 nm. Coloration was performed in accordance with the manufacturer's protocol. Objects were recorded in 530 µm areas along the Z-axis (4x lens). The stitched Z-stack images were analyzed by counting the number of cell nuclei and subsequent calculation of the number of cells per 1mm^3^.

It was found that the cell density in the scaffolds with collagen № 1 was 40% higher than in scaffolds with collagen № 2. After 72 hours, the number of cells in the scaffolds had increased by 1.23 times, which indicated the proliferative activity of the cells [4]. Dead cells identified by nuclear staining with the specific TO-PROTM3 Ready FlowTM dye in all samples were effectively absent, or only present as a few single ones in the fields of view.

## Declaration of Competing Interest

'The Authors confirm that there are no conflicts of interest.
